# Supplementary material for: eXplainable Artificial Intelligence (XAI) for improving organisational regility
Source: PLoS One. 2024 Apr 24;19(4):e0301429. doi: 10.1371/journal.pone.0301429 (PMC11042710; doi:10.1371/journal.pone.0301429)
Supplement: Attachment — Submitted filename: Respond to reviewers.docx [file pone.0301429.s001.docx]

Table 1: Reviewers’ Responses

| Item no | Response |
| --- | --- |
| Reviewer1 | We would like to thank Reviewer 1 for their excellent comments. We do appreciate your comments, they were very helpful. We have modified and restructured the mentioned sections accordingly and addressed your comments. |
| Reviewer2 | We would like to thank Reviewer 2 for their great comments. We have addressed the comments in detail within the updated version of the manuscript. |

Table 2: Detailed Reviewers’ Responses

| Item No |  |
| --- | --- |
| Reviewer 1 | Reviewer #1: eXplainable Artificial Intelligence (XAI) for improving organisational regility  1) At the end of introduction, the main contribution of the proposed study should be provided in points.  Response: Thanks for the comment. A paragraph is added to the introduction to emphasise the importance of eXplainability to this paper.  2) The quality of all the figures are very poor, authors are advised revising all the figures in 600 dpi for clear visualization. Response: The authors appreciate the time and effort of the reviewer and this constructive comment. The images have been modified and amended.  3) To show the effectiveness of the proposed study, the proposed results should be compared with the existing studies.  Response: Thanks for the comment. As outlined in the introduction, this work builds upon our previous research on prediction of organisational agility. In the previous work, we focused on the prediction concepts and algorithms.  In this work, however, we aimed the eXplainability. As you astutely mentioned in comment 1, we pinpointed the aims of the work in a nutshell to clarify it, in the introduction section. Based on the manuscripts’ results, the concept of explainability is relative and relies on the specific features we choose for our prediction problem. Therefore, It cannot be compared to other pieces of literature in this field unless all features are identical. Besides, using explainibility in the prediction of organisational ability is the main innovations of our work.  Therefore, we will add the accuracy of the random forest method as the method with the highest accuracy based on our previous study, as it is missing in the manuscript. Besides, three paragraphs are added to show the research gap, in the introduction.  4) The authors are advised to provide the hyperparameters used for training the applied machine learning models by citing the recent predictors such as DP-BINDER, pAtbP-EnC, AIPs-SnTCN, and Target-ensC_NP.  Response: The authors thank the reviewer for the deep look into the manuscript. Above mentioned parameters are related to the prediction algorithm used in previous study, and we have addressed things like hyperparameters in previous work (reference 12 of the manuscript).  5) In developing machine learning based models, the validation of the model is an important phase, the authors should make it clear that they deals with overfitting issues of the proposed study.  Response: Thank you for the comment. The focus of this work is on the explainability methods and the results demonstrated in the study are basically based on the validation sets. |
| Reviewer 2 | Reviewer #2: eXplainable Artificial Intelligence (XAI) for improving organisational regility.  1) The authors are suggested to provide up-to-date related work with critical analysis.  Response: Thanks for your comment. Three paragraphs are added to the introduction to consider some other related references and also show the gap. We also added one paragraph to the introduction to illustrate the main purpose of the paper clearly.  2) The proposed study was performed on the old data of the early 2015-16. It would be more convenient if the authors applied the methodology to the latest data.  Response: Thanks for the comment. The data used in the manuscript is not time-dependent and it has been collected from Australian business owners and it is using emerging technology. Besides, it is the continuation of the previous work.    3) The conclusion seems very lengthy and not attractive. Authors are advised to revise the conclusion section concisely and briefly.   Response: The authors want to thank the reviewer because this comment helped the manuscript’s consistency. The conclusion has been rewritten and modified.  4) What should be the real-life applications of the proposed study? Moreover, the future directions should be provided.  Response: Thanks for the comment. About the real-world application of the paper results, as stated in the conclusion, “. Our aspiration is for our findings to assume a central role in facilitating organisations’ endeavour to augment their agility, consequently empowering them to secure a competitive advantage amidst the fluid and perpetually evolving business environment”. Besides, as you wisely mentioned this issue, we added some sentences in the manuscript to clarify the importance of the manuscript’s finding in real-world application mentioning that the findings of this paper help the organisations understand the most and least important factors which affect the organisational regility and try to focus on the most important ones to improve it. Organisations can also replace the least important features with any upcoming feature during the time and optimise their resiliency and agility.  About the future direction of the work, a paragraph is added to conclusion.  5) The authors are advised to proofread the whole manuscript for typos and grammatical mistakes  Response: Thank you for the helpful comment. The whole manuscript has been grammar-checked and the issues have been addressed. |
